# Joule heating and dissipation couple effects on magneto silver-graphene hybrid nanofluids upon radial stretching surface

**DOI:** 10.1016/j.heliyon.2025.e42172

**Published:** 2025-01-26

**Authors:** M. Ragavi, P. Sreenivasulu, T. Poornima

**Affiliations:** a Department of Mathematics, School of Advanced Sciences, Vellore Institute of Technology, Vellore, 632014, India; b Department of Mathematics, Sri Venkateswara College of Engineering, Tirupati, 517507, India

**Keywords:** Keller box method, Stretching surface, Nanofluid, Joule heating, Porous medium, Convective heat transfer

## Abstract

This study aims to scrutinize the numerical exploration of the unsteady axisymmetric flow of hybrid nanofluid (Ag-Gr\H_2_O) across a radial surface. This research addresses the need for enhanced heat transfer mechanisms in industrial applications by incorporating the effects of convective thermal transfer, suction\injection, Joule heating, and viscous dissipation. The collection of flow-controlling Partial Differential Equations (PDEs) has been simplified to Ordinary Differential Equations (ODEs) by the appropriate similarity transformations. Further, the finite difference method (Keller Box technique) is incorporated to determine the numerical solutions with the assistance of MATLAB software. The fluid flow and thermal distributions are examined to understand the impact of different factors such as magnetic strength, unsteadiness, Eckert number, Biot number, suction\injection, porosity, and nanoparticle volume fraction. The results demonstrate significant enhancements in thermal distribution for the enhanced Eckert number and Biot number. As magnetic and porosity parameters increase, the flow distribution declines. Moreover, the tabular form depicts local changes in Nusselt number and skin friction coefficient for a certain range of embedded parameters. The present study was compared to prior studies and showed remarkable concurrence with previous findings. This consistency underscores the robustness of our methodology and the reliability of the results.

## Graphical abstract

**Figure:**
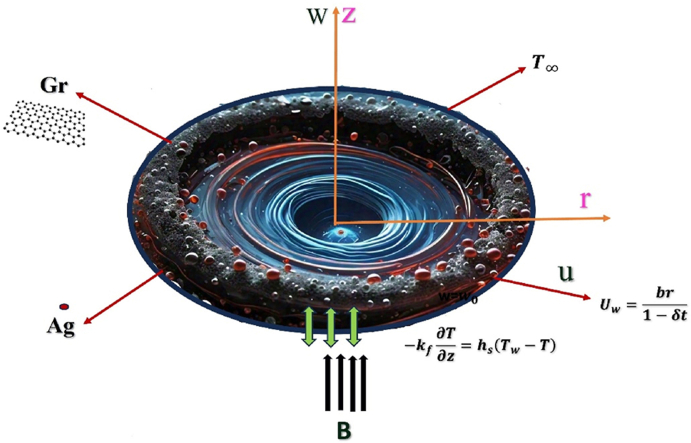

## 1 Introduction

Research on the demeanor of nanofluids (a fluid containing nanometer-sized particles) has received considerable notice in recent years, driven by their promising applications across diverse fields including engineering, biology, and medicine. Hybrid nanofluids have attracted substantial attention because of their notable enhancement in thermal conductance. Augmenting the thermal conductivity of nanofluids is presently a vital manufacturing need. An empirical investigation has shown that hybrid nanofluids are much more efficient compared to regular nanofluids. This significant increase in thermal conductivity holds the promise of substantially improving heat transfer efficiency within solar collectors. Choi [1] was the first to witness a significant increase in noble characteristics when non-metal or metal particles combine with a fluid.

Shoaib et al. [2] examine the process of diffusion and thermal transfer in a 3D magnetohydrodynamic (MHD) radiative movement of a hybrid nanofluid across a sheet that is being stretched. Khan et al. [3] analyzed the behavior of fluids that exhibit shear thinning and thickening properties in MHD (magnetohydrodynamic) flow. Hybrid nanoparticles impact on different physical properties was studied by Kumar et al. [4]. The study was carried out on a Cu-Fe_3_O_4_/ethylene glycol-based hybrid nanofluid subjected to both steady and free convection over a stretching surface. Abbas et al. [5] investigated the unstable compressible Casson hybrid nano liquid movement across a perpendicular extending surface. In their study, a comparative examination was conducted for the Yamada Ota, Tiwari Das, and Xue hybrid models. The Yamada Ota model achieves a higher heat transfer rate than other models. Shivapuji and Sulochana [6] studied the movement of a hybrid nanofluid that combines magnetohydrodynamics past a stretching sheet. Rajesh et al. [7] analyzed the heat transmission and boundary layer movement properties of a composite nanofluid (made of water/Ag-CuO) that conducts electricity. The research was conducted on a vertically stretched surface with a modified temperature. Based on thermophoresis and Brownian motion variables, Algehyne et al. [8] studied the fluid flow behavior of water-based hybrid nanoparticles across a nonlinear stretching surface.

The discussion of magnetohydrodynamics (MHD) nanofluid movements has earned considerable attention in recent times due to its importance in a territory of industrial and technical applications. Siddiqui and Shankar [9] explored the flow and thermal transfer attributes of a non-Newtonian fluid over a permeable intermediate by utilizing magnetohydrodynamics (MHD). The findings of their research have practical implications for the optimization of systems involving non-Newtonian liquids and media with sponginess. Yahkun et al. [10] conducted a study on the heat conveyance attributes of MHD hybrid nanofluids on linear stretching and shrinking surfaces, considering suction and thermal radiation effects. The study found that the thermal efficiency was higher for the hybrid nanoparticle suspension (Cu-Al_2_O_3_/H_2_O) than for the nanofluid (Cu/H_2_O). The focus of the study was to enhance thermal conductivity using a hybrid nanofluid model. Ekang et al. [11] explored the consequences of a nanofluid on heat and mass movement over an expanding surface. Srisailam et al. [12] thoroughly investigated the behavior of magnetohydrodynamic nanofluid flow on a stretching sheet. The outcomes of their study can be valuable in comprehending the behavior of nanofluids and their prospective applications. Maheswari et al. [13] focused on examining the flow of three different nanofluids (Fe_3_O_4_−H_2_O, Cu−H_2_O, and Ag−H_2_O) over a stretching surface under the influence of magnetohydrodynamics. The findings can be employed to enhance the efficiency of heat and mass transfer operations in diverse settings. Mohana and Rushikumar [14] discussed the impact of different nanoparticle morphologies on the distribution of magnetohydrodynamic boundary layers across a sheet that is being stretched. The outcome of their research revealed that nanoparticles with a platelet shape have superior flow and heat transmission capabilities.

An investigation of the viscous dissipation and Ohmic heating impacts on an MHD melting thermal transfer and boundary layer flow of a micropolar liquid over an elongating surface has been examined by Kumar et al. [15]. Shahzad et al. [16] evaluate the mobility of a tangent hyperbolic nanofluid which is non-Newtonian across a stretched surface. This research can aid in the enhancement of heat and mass transfer procedures. The impact of both viscous dissipation and joule heating on MHD three-dimensional smooth flow of a non-compressible viscous non-linear radiating Casson nanoliquid over a non-linear stretching permeable surface was investigated by Sreenivasulu et al. [17]. Ashish Mishra and Manoj Kumar [18] looked into how different aspects, including viscous-Joule heating, thermal radiation, and heat production/absorption, affected the movement of MHD nanofluid through an expanding surface that is buried in a porous medium and subject to suction/injection. Tarakaramu et al. [19] have studied the nonlinear thermal emission and Ohmic heating impacts on the MHD 3D viscoelastic nanoparticle fluid movement over a stretching surface. Their proposed framework can be implemented in the engineering and manufacturing sectors. Arifin et al. [20] studied the impact of viscous dispersion in aligned magnetohydrodynamic (MHD) movement of Jeffery fluid containing carbon nanoparticles (CNTs) across an exponential stretching surface with Newtonian heating as the boundary condition.

Patel et al. [21] used homotopy analysis method to criticize how a micropolar fluid moves through a permeable material with mixed convection. This research offers valuable insights into the implementations of industries such as polymer manufacture and extrusion. Aly and Pop [22] carried out a study on the effective analytical and numerical methods for scrutinizing the movement and thermal transfer of a magnetohydrodynamic (MHD) system. The setup used a permeable sheet and incorporated the consequences of suction and convective boundary conditions. Srisailam et al. [23] investigated the movement of MHD nanoliquid on a stretched surface near a convective boundary. Farooq et al. [24] explored the consequences of convective thermal transfer resulting from MHD nanofluid motion across a stretched surface. According to the findings of the research, nanofluids equipped with variable viscosity have the potential to enhance thermal performance in industrial mechanisms. Rashid et al. [25] explored the physical characteristics of silver and alumina nanoparticles with ethylene glycol-water impact in mixed convection micropolar fluid through the expanding surface implanted in a permeable medium.

Researchers have conducted extensive research to explore the flow of hybrid nanofluids across a radially stretched sheet. Using Casson nanofluid, Narendar et al. [26] explored the effects of radiation, magnetic flux, and heat production on the MHD (magnetohydrodynamic) stagnation point movement through a sheet that was stretched radially. Saini et al. [27] identified the time-dependent axisymmetric flow of a non-Newtonian Williamson fluid driven by a radially stretched surface with nanoparticles present in the porous medium. Resources for the study of nanofluid flow across a surface subjected to radial stretching are given in Refs. [[28], [29], [30], [31], [32]]. Ali et al. [33] investigate the nanofluid and hybrid nanofluid flow across an unsteady radially stretching sheet.

To the best of the author's knowledge, published literature has not yet attempted to use the Keller box approach for examining hybrid nanofluid flow over an unstable radial stretching surface. In this article, we investigate flow and thermal characteristics using the Keller box method, a numerical approach known for its accuracy and efficiency in solving boundary value problems. The main goal of this work is to investigate the movement of a magnetohydrodynamic (MHD) hybrid nanofluid past a convective surface implanted in a porous medium. By utilizing the appropriate similarity transformations, we convert the governing Partial Differential Equations (PDEs) into nonlinear ODEs (Ordinary Differential Equations). Further, we numerically solve these equations using the Keller box approach with the aid of MATLAB software. This study aims to provide solutions to the following research inquiries:

i. In what ways do the temperature distribution and velocity change in relation to the several flow control parameters, such as the suction/injection parameter, magnetic field, porosity parameter, Eckert number, and Biot number?
ii. What are the effects of different factors such as Eckert number, suction/injection parameter, Biot number on the coefficient of skin friction, and Nusselt numbers?
iii. How does fluid flow in the presence/absence of suction/injection over the surface?

To verify the precision and correctness of the chosen numerical technique and findings, we compared the current and previous results found in the literature.

### 1.1 Novelty

This study presents a unique investigation into the thermal and flow behavior of a hybrid nanofluid composed of silver (Ag) and graphene (Gr) nanoparticles suspended in water (H_2_O) over a radially stretching sheet considering the effects of suction/injection, convective heat transfer, Joule heating, and viscous dissipation. The innovative aspect of this study is the application of the Keller Box Technique (a finite difference method) to numerically solve the complex flow and thermal equations under these conditions.

## 2 Mathematical model

**Fig. 1:**
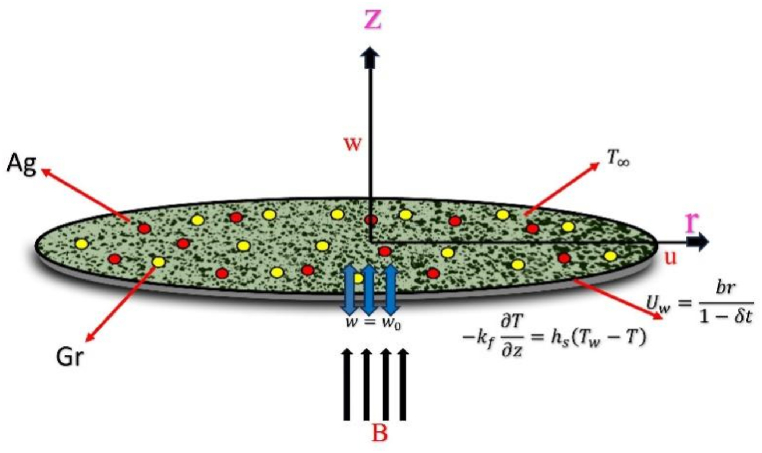
Physical model of the problem.

We examine a two-dimensional incompressible fluid movement and thermal transfer through a heated convective surface that is stretched radially, taking into account the effects of slip, Joule heating, viscous dissipation, and porous media. The sheet is aligned with the plane z = 0, indicating that the fluid is present in the region z > 0. To provide a precise mathematical description, we will utilize the cylindrical polar coordinate system (r, θ, z). For this investigation, the velocity and temperature fields are represented by the forms v = [u (r, z, t), 0, w (r, z, t)] and T = T (r, z, t). The movement phenomenon arises because the sheet is stretched in the radial direction with a velocity $U_{w}=\frac{br}{1-\delta t}$ where b and δ represent the small physical parameters associated with an unsteady stretching surface. The fluid temperature follows a specific form $T=T_{w}=T_{\infty }+T_{r}\frac{br^{2}}{\nu {\left(1-\delta t\right)}^{2}}$ , where *T*_*w*_ stands for the wall temperature and $T_{\infty }$ indicates the temperature in a free stream situation. The physical model of the problem under consideration is presented in Fig. 1.

**Assumption:** The fluid is incompressible (density remains constant), and the flow properties change over time. The flow is considered axisymmetric, indicating no variations in the angular directions. A time-dependent magnetic field $B\left(t\right)=\frac{B_{0}}{\sqrt{1-\delta t}}$ is applied along the z-axis. To enhance the heat transfer mechanism, the model includes the effects of viscous dissipation, Joule heating, and convective heat transfer.

Based on the above assumption and boundary layer approximation the governing equation for this problem can be expressed as follows [28]:

(1) $$\frac{\partial u}{\partial r}+\frac{u}{r}+\frac{\partial w}{\partial z}=0$$

(2) $$\frac{\partial u}{\partial t}+u\frac{\partial u}{\partial r}+w\frac{\partial u}{\partial z}=\frac{\mu_{hnf}}{\rho_{hnf}}\frac{\partial^{2}u}{\partial z^{2}}-\frac{\sigma_{hnf}B^{2}u}{\rho_{hnf}}-\frac{\mu_{hnf}}{\rho_{hnf}}\frac{u}{k^{*}}$$

(3) $$\frac{\partial T}{\partial t}+u\frac{\partial T}{\partial r}+w\frac{\partial T}{\partial z}=\frac{k_{hnf}}{{\left(\rho C_{p}\right)}_{hnf}}\frac{\partial^{2}T}{\partial z^{2}}+\frac{\mu_{hnf}}{{\left(\rho C_{p}\right)}_{hnf}}{\left(\frac{\partial u}{\partial z}\right)}^{2}+\frac{\sigma_{hnf}B^{2}u^{2}}{{\left(\rho C_{p}\right)}_{hnf}}$$

and the conditions at the boundary are [31].

$$u=U_{w},w=w_{0},-k_{f}\frac{\partial T}{\partial z}=h_{s}\left(T_{w}-T\right)\;\text{at}\;z=0$$

(4) $$u\to 0,T\to T_{\infty }\;\text{as}\;z\;\to \infty$$

Here, u represent the velocity components in the r direction, w represent the velocity components in the z direction, B is the magnetic field strength, and T is the temperature. Let hnf, *s*_*1*_, *s*_*2*_, *f*, $\mu_{hnf}$, $k_{hnf}$, $\sigma_{hnf}\text{,}$ $\rho_{hnf}$, $h_{s}$ denote the hybrid nanofluid, first solid particle (Ag), second solid particle (Gr), working fluid, the viscosity of hybrid nanofluid, the thermal conductivity of hybrid nanofluid, the electrical conductivity of hybrid nanofluid, density of hybrid nanofluid, and heat convection of nanofluid.Let $w_{0}=-2\sqrt{\frac{b\nu }{1-\delta t}}$ represents the mass transfer, with $w_{0}<0$ indicating suction and $w_{0}>0$ indicating injection. The thermophysical characteristics of hybrid nanofluid are described as follows [34]:

$$\frac{\mu_{hnf}}{\mu_{f}}=\frac{1}{{\left(1-\delta_{1}\right)}^{2.5}{\left(1-\delta_{2}\right)}^{2.5}}\text{,}$$

$$\rho_{hnf}=\left(1-\delta_{2}\right)\left[\left(1-\delta_{1}\right)\rho_{f}+\delta_{1}\rho_{s1}\right]+\delta_{2}\rho_{s2}\text{,}$$

$${\left({\rho C}_{p}\right)}_{hnf}=\left(1-\delta_{2}\right)\left[\left(1-\delta_{1}\right){\left({\rho C}_{p}\right)}_{f}+\delta_{1}{\left({\rho C}_{p}\right)}_{s1}\right]+\delta_{2}{\left({\rho C}_{p}\right)}_{s2}\text{,}$$

(5) $$\frac{k_{hnf}}{k_{bf}}=\frac{k_{s2}+2k_{bf}-2\delta_{2}\left(k_{bf}-k_{s2}\right)}{k_{s2}+2k_{bf}+\delta_{2}\left(k_{bf}-k_{s2}\right)},\frac{k_{bf}}{k_{f}}=\frac{k_{s1}+2k_{f}-2\delta_{1}\left(k_{f}-k_{s1}\right)}{k_{s1}+2k_{f}+\delta_{1}\left(k_{f}-k_{s1}\right)}\text{,}$$

$$\frac{\sigma_{hnf}}{\sigma_{f}}=\left(1+\frac{3\left(\frac{\sigma_{s2}}{\sigma_{f}}-1\right)\delta_{2}}{\left(\frac{\sigma_{s2}}{\sigma_{f}}+2\right)-\left(\frac{\sigma_{s2}}{\sigma_{f}}-1\right)\delta_{2}}\times 1+\frac{3\left(\frac{\sigma_{s1}}{\sigma_{f}}-1\right)\delta_{1}}{\left(\frac{\sigma_{s1}}{\sigma_{f}}+2\right)-\left(\frac{\sigma_{s1}}{\sigma_{f}}-1\right)\delta_{1}}\right)\text{.}$$

where δ_1_ and δ_2_ stand for the volume fraction of Ag and Gr nanoparticles, respectively. The similarity transformation implemented in this inquiry is exhibited as [29].

$$\psi =\frac{-r^{2}\sqrt{b\nu_{f}}}{\sqrt{1-\delta t}}f\left(\eta \right)$$

(6) $$\eta =z\sqrt{\frac{b}{\nu_{f}\left(1-t\delta \right)}}$$

$$\theta \left(\eta \right)=\frac{T-T_{\infty }}{T_{w}-T_{\infty }}$$

The subsequent clarifies the transverse and longitudinal aspects of velocity are

(7) $$u=\frac{1}{r}\frac{\partial \psi }{\partial z}=U_{w}f^{'}\left(\eta \right),w=\frac{-1}{r}\frac{\partial \psi }{\partial r}=-2\sqrt{\frac{b\nu }{1-\delta t}}\;f\left(\eta \right)\text{.}$$

Equations (8), (9), (10) provide the updated set of ODEs that result from the PDEs in equations (2), (3), (4).

(8) $${B_{1}f}^{\prime \prime \prime }+2ff^{\prime \prime }-{\left(f^{\prime }\right)}^{2}-J_{s}\left(f^{\prime }+\frac{\eta }{2}f^{\prime \prime }\right)-B_{2}M_{n}f^{\prime }-B_{1}D_{k}f^{\prime }=0\text{,}$$

(9) $$\frac{{B_{3}\theta }^{''}}{\mathrm{Pr}}+2f\theta^{'}-2f^{'}\theta +B_{4}E_{k}{\left(f^{''}\right)}^{2}-J_{s}\left(2\theta +{\frac{\eta }{2}\theta }^{'}\right)+B_{5}M_{n}E_{k}{\left(f^{'}\right)}^{2}=0\text{,}$$

Now, the boundary condition (4) transforms into

$$f^{\prime }\left(\eta \right)=1,{\;f\left(\eta \right)=S_{c},\theta }^{\prime }\left(\eta \right)=-B_{t}\left(1-\theta \left(\eta \right)\right)\;\text{at}\;\eta =0\text{,}$$

(10) $$f^{\prime }\left(\eta \right)\to 0,\theta \left(\eta \right)\to 0\;as\;\eta \to \infty \text{.}$$

The variables B_1_, B_2_, B_3_ B_4_, and B_5_ in the equation shown above are explicitly defined as follows:

B1=μhnfμfρhnfρf,B2=σhnfσfρhnfρf,B3=khnfkf(ρCp)hnf(ρCp)f,B4=μhnfμf(ρCp)hnf(ρCp)f,B5=σhnfσf(ρCp)hnf(ρCp)f.

Here are a few constants that do not have dimensions: $E_{k}=\frac{b\nu_{f}}{C_{p}\left(T_{r}\right)}$ is the Eckert number; $J_{s}=\frac{\delta }{b}$ represents the unsteadiness parameter; $\mathrm{Pr}=\frac{\rho C_{p}\nu_{f}}{k_{f}}$ is the Prandtl number; $M_{n}=\frac{B_{0}^{2}\sigma_{f}}{b\rho_{f}}$ is the magnetic parameter; $B_{t}=\frac{h_{0}}{k}\sqrt{\frac{\nu_{f}}{b}}$ is the Biot number; ${Re}_{r}=\frac{rU_{w}}{\nu_{f}}$ is the Reynolds number; $D_{k}=\frac{\left(1-\delta t\right)\mu_{f}}{\rho_{f}{bK}^{*}}$ is the porosity parameter, $S_{c}>0$ indicates suction and $S_{c}<0$ indicates injection. The thermophysical properties of nanoparticles and base fluid are listed in Table 1.

**Table 1:** Properties of nanoparticles and base fluid [35].

Physical characters	Ag	Gr	H_2_O
k (*W/mK*)	429	2500	0.613
*σ* (Ωm)^−1^	6.3 × 10^7^	1 × 10^7^	0.05
*C*_*p*_ (*J/kgK*)	235	2100	4179
*ρ* (*kg/m*^3^)	10,500	2250	997.1

The key physical parameters relevant to the governing flow problem include the local shear rate (C_f_), and Nusselt number (*Nu*) which are designated as [29].

(11) $$C_{f}=\frac{\tau_{w}}{\rho_{f}{U_{w}}^{2}},Nu=\frac{rq_{w}}{k_{f}\left(T_{w}-T_{\infty }\right)}\;\text{at}\;z=0\text{.}$$

Where $\tau_{w}=\mu_{hnf}\left[\frac{\partial u}{\partial z}\right],and\;q_{w}=-k_{hnf}\frac{\partial T}{\partial z}$

The shear rate coefficient and heat flow at the boundary are represented by the values illustrated above. Equation (11) can be simplified to the following form:

$$C_{f}\sqrt{Re}={\frac{\mu_{hnf}}{\mu_{f}}f}^{\prime \prime }\left(0\right)\text{,}$$

(12) Re−12Nu=−khnfkfθ′(0).

## 3 Numerical procedure

Through the implementation of transformations, the set of differential systems (1–3) together with boundary condition (4) is changed into an ordinary differential equation. In this scenario, the finite difference approach (Keller Box method) is a numerical technique chosen for solving boundary value problems. The method involves discretizing the domain into a grid and iteratively solving the equations at each grid point until a convergence criterion is met. To obtain the solutions, the following steps can be taken:

• Make the differential equation system into a system of first-order equations.
• Representing equations in finite difference form by implementing centered-difference approximations.
• Incorporate Newton's linearization algorithm into the equations (if nonlinear).
• Utilize the Block-Tridiagonal-Elimination technique to compute the solution of the reduced system.

It is required to add a new dependent variable (l, p, t) in order to formulate equations in a first-order system.

(13) $$f^{\prime }=l,l^{\prime }=p,\theta^{\prime }=t\text{.}$$

Then,

(14) $$B_{1}p^{\prime }+2fp-{\left(l\right)}^{2}-J_{s}\left(l+\frac{\eta }{2}p\right)-l\left({B_{2}M}_{n}+B_{1}D_{k}\right)=0\text{,}$$

(15) $$B_{3}\frac{t^{'}}{\mathrm{Pr}}+2ft-2l\theta +{B_{4}E}_{k}{\left(p\right)}^{2}-J_{s}\left(2\theta +\frac{\eta }{2}t\right)+B_{5}M_{n}E_{k}l^{2}=0\text{,}$$

the boundary condition turns into

$$f\left(\eta \right)=S_{c},l\left(\eta \right)=1,t\left(\eta \right)=-B_{t}\left(1-\theta \left(\eta \right)\right)\;\text{at}\;\eta =0\text{,}$$

(16) l(η)=0,θ(η)=0asη→∞.

i) Finite difference scheme:

The implementation of the finite difference technique to Equations (13), (14), (15), (16) results in the following system of equations:

(17) $$f_{j}-f_{j-1}-\frac{h_{j}}{2}\left(l_{j}+l_{j-1}\right)=0\text{.}$$

(18) $$l_{j}-l_{j-1}-\frac{h_{j}}{2}\left(p_{j}+p_{j-1}\right)=0\text{.}$$

(19) $$\theta_{j}-\theta_{j-1}-\frac{h_{j}}{2}\left(t_{j}+t_{j-1}\right)=0\text{.}$$

(20) $$B_{1}\left(p_{j}-p_{j-1}\right)+2\frac{h_{j}}{2}\left(f_{j}+f_{j-1}\right)\left(p_{j}+p_{j-1}\right)-\frac{h_{j}}{2}{\left(l_{j}+l_{j-1}\right)}^{2}-\frac{h_{j}}{2}J_{s}\left[\left(l_{j}+l_{j-1}\right)+\frac{\eta }{2}\left(p_{j}+p_{j-1}\right)\right]-\frac{h_{j}}{2}\left(l_{j}+l_{j-1}\right)\left({B_{2}M}_{n}+B_{1}D_{k}\right)=0\text{.}$$

(21) $$\frac{B_{3}}{\mathrm{Pr}}\left[t_{j}-t_{j-1}\right]+2\frac{h_{j}}{2}\left(f_{j}+f_{j-1}\right)\left(t_{j}+t_{j-1}\right)-2\frac{h_{j}}{2}\left(l_{j}+l_{j-1}\right)\left(\theta_{j}+\theta_{j-1}\right)+B_{4}E_{k}\frac{hj}{2}{\left(p_{j}+p_{j-1}\right)}^{2}-J_{s}\frac{h_{j}}{2}\left[2\left(\theta_{j}+\theta_{j-1}\right)+\frac{\eta }{2}\left(t_{j}+t_{j-1}\right)\right]+B_{5}M_{n}E_{k}\frac{hj}{2}{\left(l_{j}+l_{j-1}\right)}^{2}=0\text{.}$$

These equations are enforced for *j* = 1, 2, 3, …...J − 1, and the boundary conditions at j = 0 and j = J are

$$f_{0}=S_{c},l_{0}=1,t_{0}=-B_{t}\left(1-\theta_{0}\right)\text{,}$$

(22) $$l_{J}=0,\theta_{J}=0\text{.}$$

ii) Newton's linearization method:

To deal with nonlinear equation 17–21, We adopted Newton's approach and presented a subsequent $\left[f_{j}^{i},l_{j}^{i},{\;p}_{j}^{i},\theta_{j}^{i},{\;t}_{j}^{i}\right]$ where i = 0, 1, 2, … For further iterations, we determined the following:

$$f_{j}^{i+1}=f_{j}^{i}{+\delta f}_{j}^{i},l_{j}^{i+1}=l_{j}^{i}{+\delta l}_{j}^{i},p_{j}^{i+1}=p_{j}^{i}{+\delta p}_{j}^{i}\text{,}$$

(23) $$\theta_{j}^{i+1}=\theta_{j}^{i}{+\delta \theta }_{j}^{i},t_{j}^{i+1}=t_{j}^{i}{+\delta }_{j}^{i}\;\text{.}$$

Subsequently, we inserted the right-hand side of Equations (17), (18), (19), (20), (21) and neglected the components of higher order in δ. The formulation of linear equations is as follows (the superscript *i* has been removed for simplicity):

$$\delta f_{j}-\delta f_{j-1}-\frac{h_{j}}{2}\left(\delta l_{j}+{\delta l}_{j-1}\right)={\left(r_{1}\right)}_{j,}$$

$$\delta l_{j}-\delta l_{j-1}-\frac{h_{j}}{2}\left(\delta p_{j}{+\delta p}_{j-1}\right)={\left(r_{2}\right)}_{j}\text{,}$$

(24) $$\delta \theta_{j}-\delta \theta_{j-1}-\frac{h_{j}}{2}\left(\delta q_{j}+{\delta q}_{j-1}\right)={\left(r_{3}\right)}_{j}\text{,}$$

$${\left(a_{1}\right)}_{j}\delta p_{j}+{\left(a_{2}\right)}_{j}{\delta p}_{j-1}+{\left(a_{3}\right)}_{j}\delta l_{j}+{\left(a_{4}\right)}_{j}{\delta l}_{j-1}+{\left(a_{5}\right)}_{j}\delta f_{j}+{\left(a_{6}\right)}_{j}\delta f_{j-1}={\left(r_{4}\right)}_{j}\text{,}$$

$${\left(b_{1}\right)}_{j}\delta t_{j}+{\left(b_{2}\right)}_{j}{\delta t}_{j-1}+{\left(b_{3}\right)}_{j}\delta f_{j}+{\left(b_{4}\right)}_{j}{\delta f}_{j-1}+{{\left(b_{5}\right)}_{j}\delta \theta }_{j}+{\left(b_{6}\right)}_{j}{\delta \theta }_{j-1}+{\left(b_{7}\right)}_{j}\delta p_{j}+{\left(b_{8}\right)}_{j}\delta p_{j-1}+{\left(b_{9}\right)}_{j}\delta l_{j}+{\left(b_{10}\right)}_{j}\delta l_{j-1}={\left(r_{5}\right)}_{j}\text{.}$$

Where,

$${\left(a_{1}\right)}_{j}=B_{1}+h_{j}f_{j-\frac{1}{2}}-\frac{h_{j}}{2}\frac{\eta }{2}J_{s}\text{,}$$

$${\left(a_{2}\right)}_{j}={\left(a_{1}\right)}_{j}-{2B}_{1}\text{,}$$

$${\left(a_{3}\right)}_{j}=h_{j}p_{j-\frac{1}{2}},{\left(a_{4}\right)}_{j}={\left(a_{3}\right)}_{j}\text{,}$$

$${\left(a_{5}\right)}_{j}=-h_{j}l_{j-\frac{1}{2}}-\frac{h_{j}}{2}J_{s}-\frac{h_{j}}{2}\left({B_{2}M}_{n}+B_{1}D_{k}\right),{\left(a_{6}\right)}_{j}={\left(a_{5}\right)}_{j,}$$

$${\left(b_{1}\right)}_{j}=\frac{B_{3}}{\mathrm{Pr}}+\frac{2h_{j}}{2}f_{j-\frac{1}{2}}-\frac{h_{j}}{2}\frac{\eta }{2}J_{s},{\left(b_{2}\right)}_{j}=-\frac{B_{3}}{\mathrm{Pr}}+\frac{2h_{j}}{2}f_{j-\frac{1}{2}}-\frac{h_{j}}{2}\frac{\eta }{2}J_{s}\text{,}$$

$${\left(b_{3}\right)}_{j}=\frac{2h_{j}}{2}t_{j-\frac{1}{2}},{\;\left(b_{4}\right)}_{j}={\left(b_{3}\right)}_{j}\text{,}$$

$${\left(b_{5}\right)}_{j}=-J_{s}\frac{h_{j}}{2}-2\frac{h_{j}}{4}l_{j-\frac{1}{2}},{\left(b_{6}\right)}_{j}={\left(b_{5\;}\right)}_{j},{\left(b_{7}\right)}_{j}={-2\frac{h_{j}}{4}\theta }_{j-\frac{1}{2}}+M_{n}B_{5}E_{k}\frac{h_{j}}{2}l_{j-\frac{1}{2}},{\left(b_{8}\right)}_{j}={\left(b_{7}\right)}_{j}\text{,}$$

$${\left(b_{9}\right)}_{j}={B_{4}E}_{k}\frac{h_{j}}{2}p_{j-\frac{1}{2}},{\left(b_{10}\right)}_{j}={\left(b_{9}\right)}_{j}\text{,}$$

$${\left(r_{1}\right)}_{j}=f_{j-1}-f_{j}+h_{j}l_{j-\frac{1}{2}},{\;\left(r_{2}\right)}_{j}=l_{j-1}-l_{j}+h_{j}p_{j-\frac{1}{2}},{\left(r_{3}\right)}_{j}=\theta_{j-1}-\theta_{j}+h_{j}q_{j-\frac{1}{2}}\text{,}$$

$${\left(r_{4}\right)}_{j}=B_{1}\left(p_{j-1}-p_{j}\right)-2h_{j}f_{j-\frac{1}{2}}p_{j-\frac{1}{2}}+h_{j}{\left(l_{j-\frac{1}{2}}\right)}^{2}+J_{s}h_{j}\left[l_{j-\frac{1}{2}}+\frac{\eta }{2}p_{j-\frac{1}{2}}\right]+h_{j}l_{j-\frac{1}{2}}\left({B_{2}M}_{n}+B_{1}D_{k}\right)\text{,}$$

$${\left(r_{5}\right)}_{j}=\frac{B_{3}}{\mathrm{Pr}}\left(t_{j-1}-t_{j}\right)-2h_{j}f_{j-\frac{1}{2}}t_{j-\frac{1}{2}}+2h_{j}l_{j-\frac{1}{2}}\theta_{j-\frac{1}{2}}-E_{k}B_{4}h_{j}{\left(p_{j-\frac{1}{2}}\right)}^{2}-B_{5}M_{n}E_{k}{\left(l_{j-\frac{1}{2}}\right)}^{2}h_{j}+J_{s}\left[2\theta_{j-\frac{1}{2}}+\frac{\eta }{2}t_{j-\frac{1}{2}}\right]h_{j.}$$

iii) Block tridiagonal structure:

The matrix representation of the linearized set of equation (24) appears as follows:

$$\left[\begin{array}{cccccccc} \left[A_{1}\right] & \left[C_{1}\right] & & & & & & \\ \left[B_{2}\right] & \left[A_{2}\right]\; & \;\left[C_{2}\right] & & & & & \\ & \left[B_{3}\right] & \left[A_{3}\right]\; & \left[C_{3}\right] & & & & \\ & & & & \ddots & \ldots & & \\ & & & & & \left[B_{j-1}\right] & \left[A_{j-1}\right] & \left[C_{j-1}\right]\\ & & & & & & \left[B_{j}\right] & \left[A_{j}\right] \end{array}\right]\left[\begin{array}{c} \left[\delta_{1}\right]\\ \left[\delta_{2}\right]\\ \left[\delta_{3}\right]\\ \vdots \\ \left[\delta_{j-1}\right]\\ \left[\delta_{J}\right] \end{array}\right]=\left[\begin{array}{c} \left[r_{1}\right]\\ \left[r_{2}\right]\\ \left[r_{3}\right]\\ \vdots \\ \left[r_{j-1}\right]\\ \left[r_{j}\right] \end{array}\right]$$

or

(25) [A][δ]=[r]

(26) A1=00100−h200−h200−h200−h2a210a31a110b101b21b31b91b11,h=hj

for 2≤j≤J,

(27) [Aj]=[−h20100−100−h200−100−h2(a6)j0(a3)j(a1)j0(b8)j(b6)j(b3)j(b9)j(b1)j]

(28) [Bj]=[00−100000−h200000−h200(a4)j(a2)j000(b4)j(b10)j(b2)j]

(29) [Cj]=[−h200001000001000(a5)j0000(b7)j(b5)j000]

In the next step, we will solve Equation (25) by assuming A is a non-singular matrix and use LU decomposition to factor it.

(30) [A]=[L][U]

Where,

(31) $$\left[L\right]=\left[\begin{array}{ccccc} \left[\alpha_{1}\right] & & & & \\ \left[\beta_{2}\right] & \left[\alpha_{2}\right] & & & \\ & & \ddots & & \\ & & & \left[\alpha_{J-1}\right] & \\ & & & \left[\beta_{J}\right] & \left[\alpha_{J}\right] \end{array}\right],\left[U\right]=\left[\begin{array}{ccccc} \left[I_{1}\right] & \left[\gamma_{1}\right] & & & \\ & [I_{2]} & \left[\gamma_{2}\right] & & \\ & & \ddots & & \\ & & & \left[I_{J-1}\right] & \left[\gamma_{J-1}\right]\\ & & & & \left[I_{J}\right] \end{array}\right]$$

These $[\alpha_{j}],[\beta_{j}$], $\left[\gamma_{j}\right]$ are grouped in 5 × 5 matrices, with their values determined by the following equations:

Equation (31) is plugged into (25), yielding

(32) [L][U][δ]=[r].

Let

(33) [U][δ]=[S].

Then,

(34) [L][S]=[r].

where,

(35) $$\left[S\right]=\left[\begin{array}{c} \left[S_{1}\right]\\ \left[S_{2}\right]\\ \vdots \\ \left[S_{J-1}\right]\\ \left[S_{J}\right] \end{array}\right]$$

In this case, $\left[S_{j}\right]$ denotes a 5 × 1 column matrix structure, with elements that may be found by solving Equation (32) including:

(36) $$\left[\alpha_{1}\right]\left[S_{1}\right]=\left[r_{1}\right],\left[\alpha_{j}\right]\left[S_{j}\right]=\left[r_{j}-{\;\beta }_{j}\right]\left[S_{j-1}\right],2\leq j\leq J\;\text{.}$$

The values of $\gamma_{j}$, $\alpha_{j}$, and $S_{j}$ are determined through a forward sweep. Subsequently, the elements of δ can be easily obtained from Equation (33) using a backward sweep, resulting in the following elements are

(37) $$\left[\delta_{J}\right]=\left[S_{J}\right],\left[\delta_{j}\right]=\left[S_{j}\right]-\left[\gamma_{j}\right]\left[\delta_{j+1}\right],1\leq j\leq J-1\;\text{.}$$

The processes of iteration are executed repeatedly until the desired level of precision $\left|\delta v_{o}^{\left(i\right)}\right|<\epsilon$ is attained, where ϵ is a small prescribed value. The convergence criterion of ϵ=0.0001 or 10^−5^ achieves an accuracy of four decimal places. All calculated solutions demonstrated a tolerance of less than 10^−6^.

## 4 Findings and analysis

This section discusses the impact of various parameters on thermal and flow characteristics via graphical and numerical data. The fluid velocity and temperature fluctuation are scrutinized concerning the values of the solid volume fraction ($\delta_{1}{=\delta }_{2}=0.02$), local Biot number (B_t_ = 0.5), porosity parameter (D_k_ = 0.5), unsteadiness parameter (J_s_ = 0.2), magnetic parameter (M_n_ = 0.2), suction (S_c_ = 0.2), injection (S_c_ = −0.2), Prandtl number (Pr = 6.2), and Eckert number (E_k_ = 0.2). The parameter ranges for Biot number (0.5≤ B_t_ ≤ 2), unsteadiness parameter (0.02 ≤ J_s_ ≤ 0.08), Eckert number (0.1 ≤ E_k_ ≤ 0.5), and magnetic parameter (0.2 ≤ M_n_ ≤ 0.8). When dealing with one parameter, we assume that all other parameters remain constant. Additionally, the computational results for the skin friction coefficient (wall shear stress) and the Nusselt number (wall thermal flux) are thoroughly examined. In order to validate our study, we have presented the current result along with the existing literature in Table 2. The current findings show a high level of concurrence with the prior findings.

**Table 2:** Validation of our findings when $D_{k}=\delta_{1}{=M_{n}=\delta }_{2}=0.0$

J_s_	S_c_	[27]	Current result
0.5	−1	−0.620400	−0.620408
	−0.5	−0.887200	−0.887201
	1	−2.655999	−2.655999
0	0.5	−1.798999	−1.798999

### 4.1 Velocity profile

**Fig. 2:**
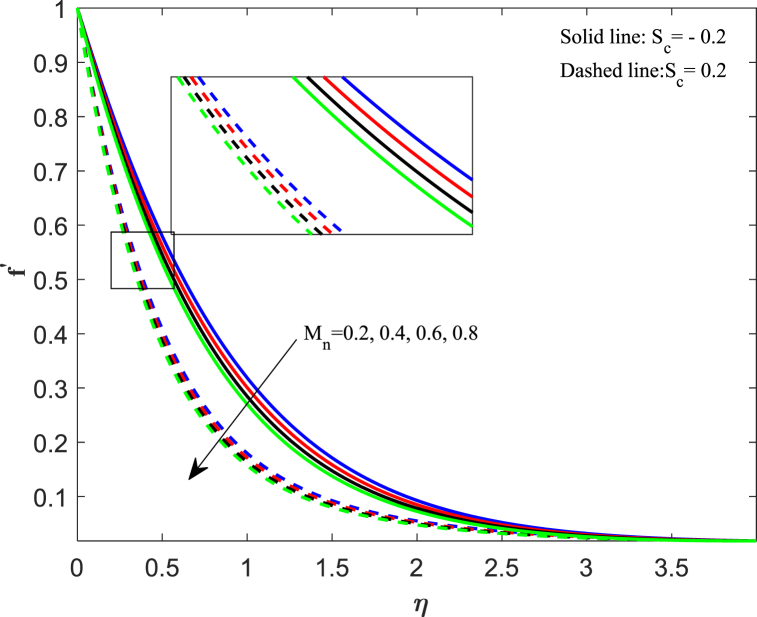
Impact of magnetic parameter (*M*_*n*_) on velocity distribution.

**Fig. 3:**
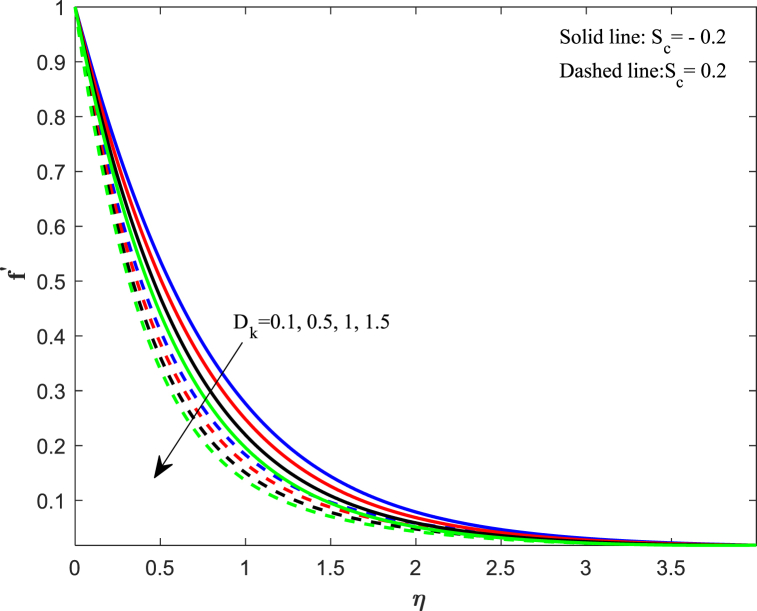
Impact of porosity parameter (*D*_*k*_) on velocity distribution.

**Fig. 4:**
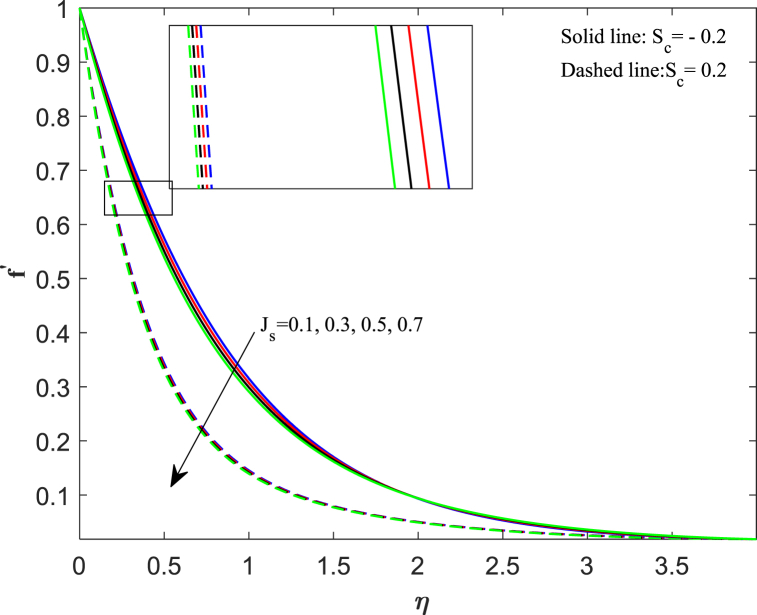
Impact of unsteadiness parameter (*J*_*s*_) on velocity distribution.

**Fig. 5:**
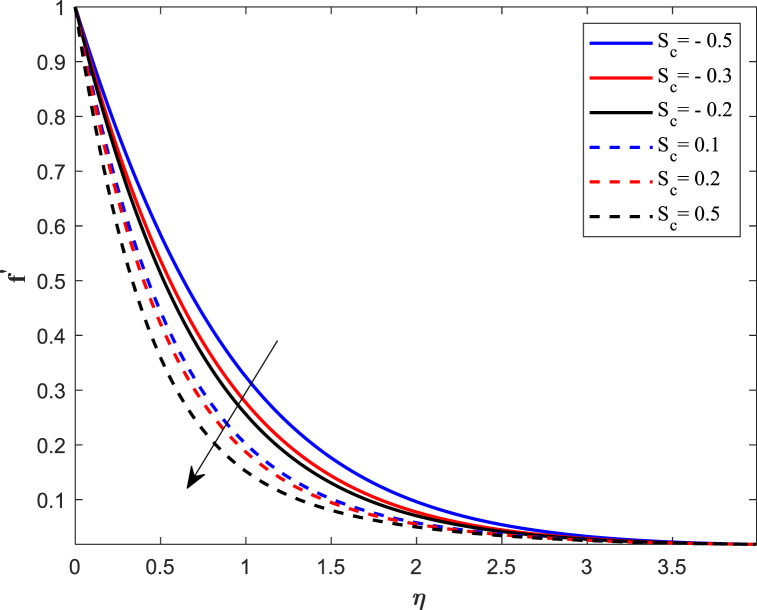
Impact of suction and injection parameter (*S*_*c*_) on velocity distribution.

The distribution of velocity completely determines the flow of fluid onto a specific surface. The analysis of fluid flow rate changes relies heavily on this particular factor. The behavior of fluid changes when subjected to external forces, and one such sort of external force is the magnetic field. Fig. 2 illustrates the impact of the magnetic parameter (M_n_) on the distribution of fluid velocity $f^{\prime }(\eta$). The distribution of velocities demonstrates an inverse relationship with the magnetic field. The velocity of fluid flattens as the magnetic parameter value increases for both suction and injection cases. When a magnetic field interacts with a flowing fluid, it stimulates the fluid particles, resulting in a counterforce that slows down and decreases the movement of fluid. On the other side, the Lorentz force, which was initially a resistive force, emphasizes that increasing the magnetic field lowers the thickness of the boundary layer. Fig. 3 displays the impact of the porosity parameter (D_k_) on the velocity distribution. We observe that larger variations in the porosity parameter lead to a decrease in the velocity profile for both cases. Higher values of the porosity parameter imply an increase in viscous forces, resulting in a dominance of inertial forces, which subsequently leads to a reduction in velocity. The velocity distribution response to changes in the unsteadiness parameter (J_s_) is illustrated in Fig. 4. Upon analyzing the findings, we noticed a decrease in velocity behavior as the unsteadiness parameter ($J_{s}$) increased. In these conditions, the thickness of the boundary layer linked to the velocity gradient reduces. When a fluid flow becomes unstable, it can lead to flow instabilities, which affect its predictability and may result in oscillations. Unsteadiness in the flow causes this instability, disrupting the fluid smooth movement and causing a decrease in its speed. Fig. 5 reveals the implications of the suction/injection (S_c_) parameter on the flow characteristics of the nanofluid. The velocity of the fluid near the surface experiences a gradual decrease as the suction parameter increases, whereas it increases for the injection case. In terms of physical behavior, the suction effect exerts a drag force on the fluid. As the quantity of fluid surrounding the sheet diminishes, the remaining fluid encounters progressively higher levels of resistance to its flow. Therefore, the velocity distribution progressively decreases.

### 4.2 Temperature profile

**Fig. 6:**
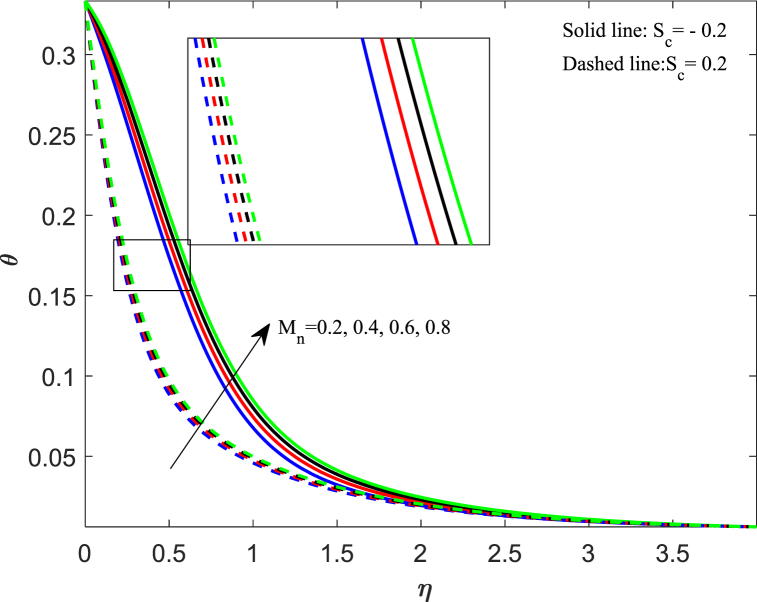
Impact of magnetic parameter (*M*_*n*_) on temperature distribution.

**Fig. 7:**
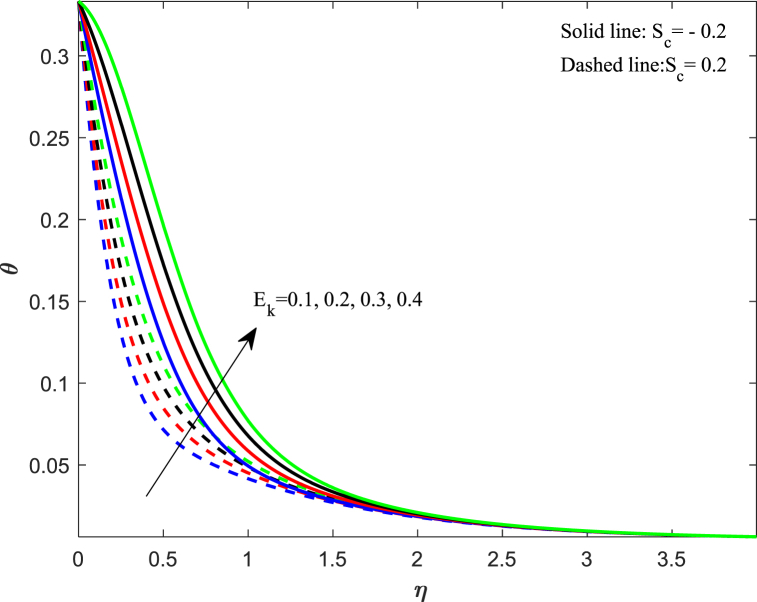
Impact of Eckert number (*E*_*k*_) on temperature distribution.

**Fig. 8:**
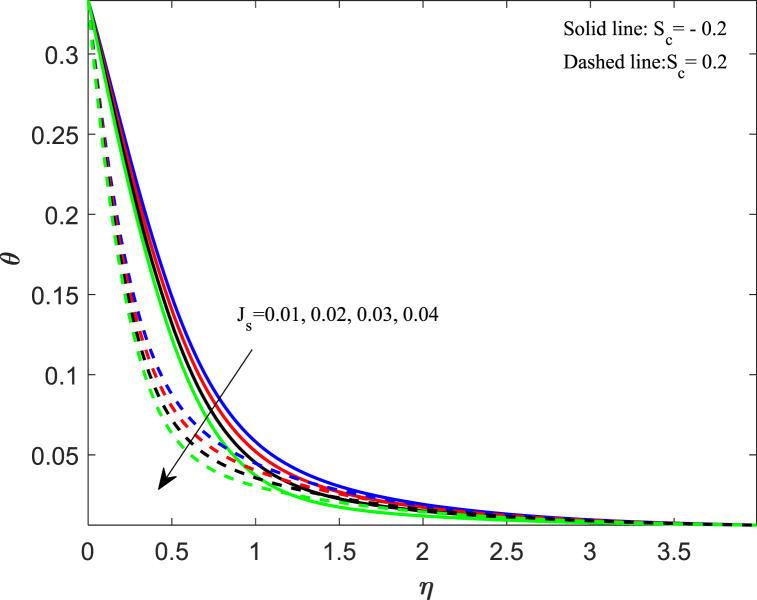
Impact of unsteadiness parameter (*J*_*s*_) on temperature distribution.

**Fig. 9:**
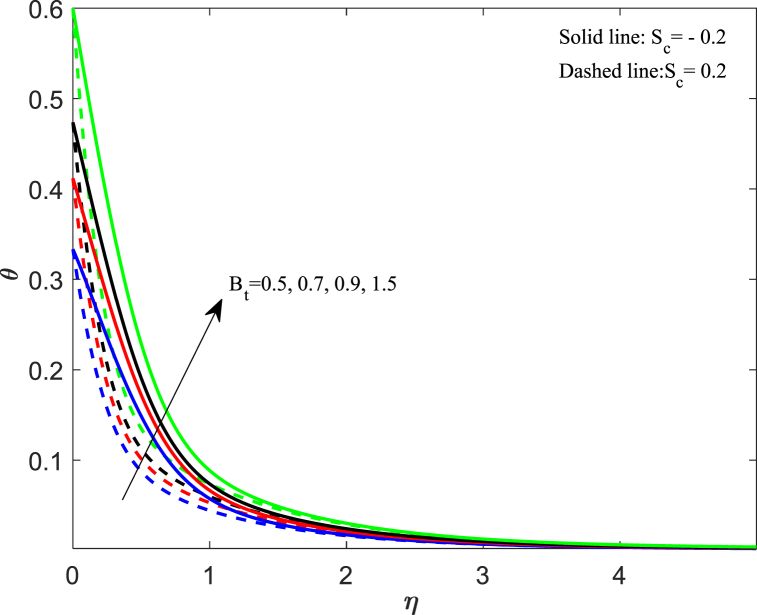
Impact of Biot number (*B*_*t*_) on temperature distribution.

The fluctuation in the temperature of the fluid has a significant impact on its behavior as well as on the particles within it. In this section, we discussed how different physical parameters impact the distribution of fluid temperature. The information conveyed in Fig. 6 indicates that when the magnetic parameter (M_n_) increases, the temperature profile increases gradually for both cases. The magnetic force creates resistance among fluid particles, leading to the production of thermal energy and a subsequent rise in temperature within the boundary layer. The effects of changing the Eckert number (E_k_) on the thermal profile are demonstrated in Fig. 7. Our observation shows that an increase in the Eckert number leads to higher thermal distribution. The Eckert number is a measure of the relationship between kinetic energy and enthalpy, which represents the process of converting kinetic energy to the form of internal energy with the aid of work done against the viscous fluid stresses. When the value of E_k_ is high, the fluid molecules undergo vibration and collision due to high kinetic power. This leads to a rise in the thermal distribution and a greater dissipation of heat in the boundary layer area. In Fig. 8, the impact of the unsteadiness parameter (J_s_) on the distribution of temperature can be observed. It shows that the temperature decreases significantly as the unsteadiness parameters increase. The rate at which heat transfers from the sheet to the fluid reduces, resulting in a reduction in temperature. The implications of Biot number (B_t_) on the thermal profile are seen in Fig. 9. From the analysis, we illustrate that the dimensionless thermal distribution grows in proportion to the rise in B_t_. While the strength of convection is heightened, it leads to an elevation in the temperature of the surface, thereby allowing the thermal effect to extend further into the fluid. The utilization of nanofluid with convective boundary conditions presents a more fitting model when compared to constant surface temperature conditions.

### 4.3 Skin friction and local Nusselt number

Table 3 exhibits the numerical values of distinct physical parameters, such as the suction/injection parameter (S_c_), unsteadiness parameter (J_s_), porosity parameter (D_k_), and magnetic parameter (M_n_), on surface shear stress. The investigation reveals that as the values of S_c_, J_s_, D_k_, and M_n_ increase, the skin friction decreases. Table 4 presents the numerical values for the impact of distinct parameters such as the magnetic parameter (M_n_), Eckert number (E_k_), Biot number (B_t_), and unsteadiness parameter (J_s_) on surface heat flux. The Nusselt number descends with rising values of M_n_, E_k_, while rising values of J_s_ and B_t_ show the opposite trend.

**Table 3:** Numerical values of skin friction coefficient for different parameters.

M_n_	D_k_	J_s_	C_f_${Re}_{r}$^1/2^	
			Suction	Injection
0.2	0.5	0.02	−1.774556	−1.332295
0.4			−1.839586	−1.396035
0.6			−1.902481	−1.457652
0.8			−1.963407	−1.517322
0.2	0.1		−1.645172	−1.205318
	0.5		−1.774556	−1.332295
	1		−1.924739	−1.479453
	1.5		−2.064218	−1.616039
		0.02	−1.721261	−1.278262
	0.5	0.04	−1.727251	−1.284339
		0.06	−1.733224	−1.290397
		0.08	−1.739180	−1.296437

**Table 4:** Numerical values of Nusselt number for different parameters.

M_n_	E_k_	J_s_	B_t_	*Nu*${Re}_{r}$^−1/2^	
				Suction	Injection
0.2	0.2	0.02	0.5	0.423004	0.367061
0.4				0.414743	0.352172
0.6				0.406726	0.337536
0.8				0.398931	0.323128
0.2	0.1			0.465496	0.410714
	0.5			0.423004	0.367061
	1			0.380515	0.323408
	1.5			0.338024	0.279755
	0.2	0.02		0.420747	0.358125
		0.04		0.421005	0.359174
		0.06		0.421262	0.360209
		0.08		0.421517	0.361230
		0.02	0.5	0.423004	0.367061
			1	0.770778	0.615434
			1.5	1.061751	0.794672
			2	1.308787	0.930115

### 4.4 Streamlines

Streamlines offer a clear and concise way to illustrate the behavior of fluid flow. It is possible to track the movement of nanofluid in the vicinity of the stretching surface for various values of suction/injection through streamlines. Here we discussed the different cases:

a) Suction case (S_c_ = 0.5,1.0,2.0)
b) Injection case (S_c_ = −0.5,−1.0,−2.0)
c) Absence of Suction/injection (S_c_ = 0)

**Fig. 10:**
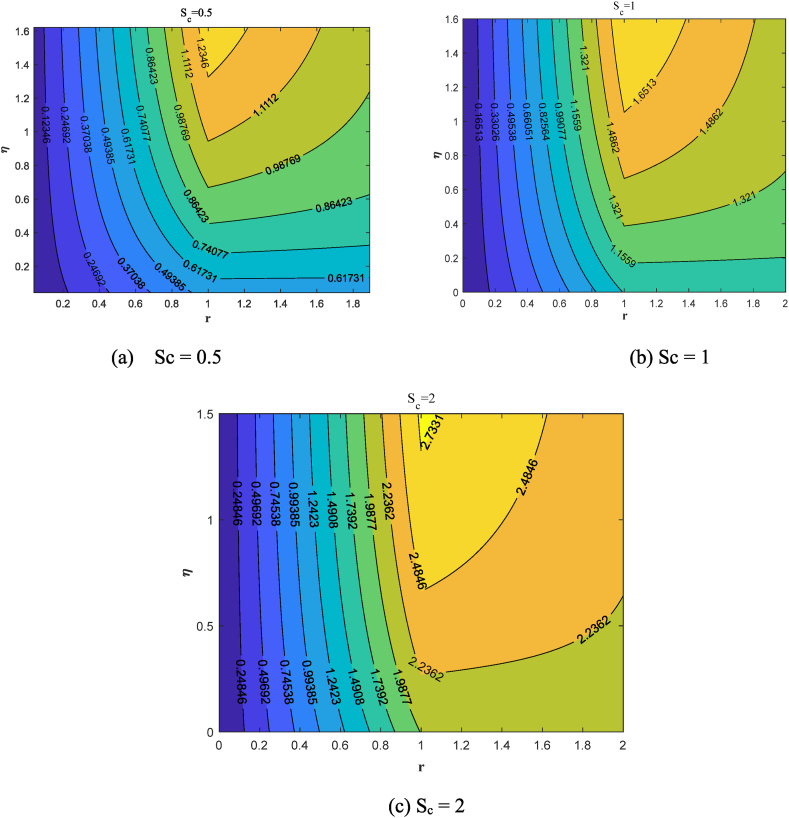
Streamlines pattern for the presence of suction (S_c_ = 0.5, 1, 2).

**Fig. 11:**
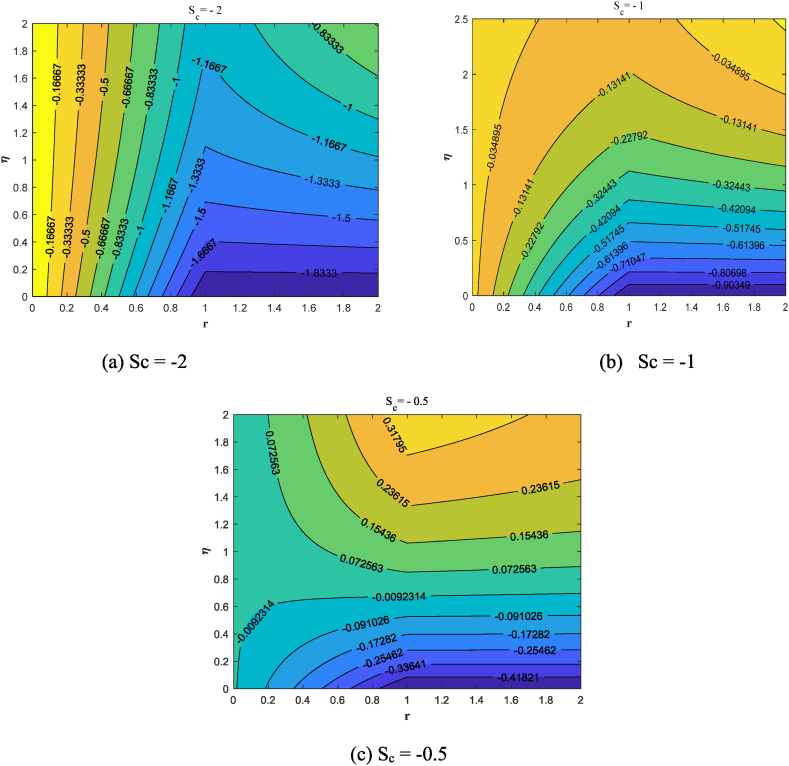
Streamlines pattern for injection (S_c_ = −0.5,-1,-2).

**Fig. 12:**
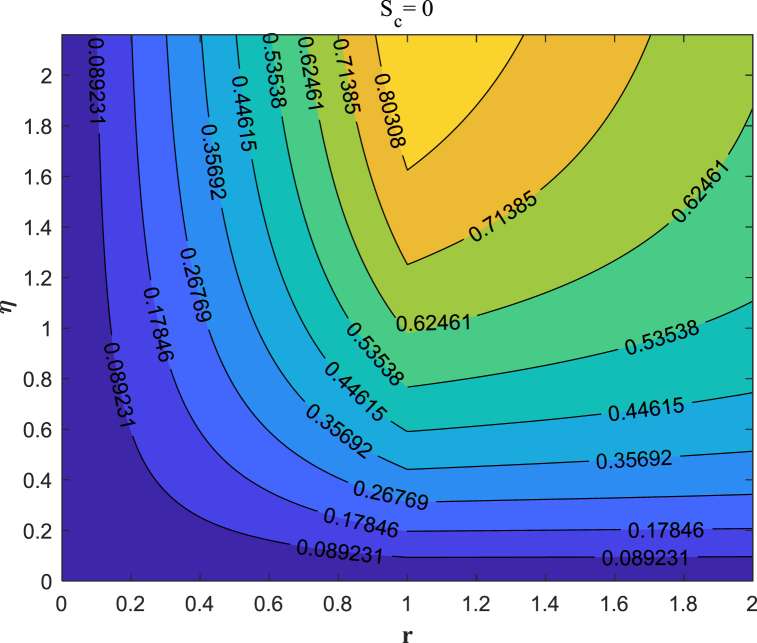
Streamlines pattern for the absence of suction.

A suction flow is applied perpendicular to the sheet, drawing fluid towards the surface. Fig. 10(a–c) displays the streamlines contour illustrating the flow of fluid over the surface in the suction (S_c_ = 0.5,1.0,2.0) situation. Suction removes fluid from the boundary layer, causing it to become thinner.The reduced boundary layer thickness causes streamlines to converge towards the sheet. Fig. 11(a–c) demonstrates how fluid flows over the surface with injection (S_c_ = −2.0,−1.0,−0.5) case. Curved Streamlines show the combined effect of radial and injection flows, indicating regions of shear and mixing. Injection adds fluid to the boundary layer, making it thicker. The increased boundary layer thickness causes streamlines to diverge away from the sheet.In Fig. 12, we study the dynamics of fluid flow in the absence of suction/injection (S_c_ = 0).

## 5 Conclusion

The destination of this study is to analyze the behaviors of a hybrid nanofluid movement over an unstable radially stretched surface, taking into account convective heat transfer and the significance of suction/injection. Additionally, we incorporate Ohmic heating, viscous dissipation, and porosity to investigate flow fluctuation and thermal transmission. To simplify the equations, we transform the partial differential equations (PDE) into ordinary differential equations (ODE) by employing similarity variables. Next, we implement the Keller Box method (finite difference method) in MATLAB software to obtain numerical solutions. The primary results derived from this study can be succinctly summarized as follows:

• A corresponding reduction in the velocity profile is observed as the magnetic, porosity, and unsteadiness parameters increase.
• The temperature distribution over the surface increases with the enhancement of magnetic, Eckert, and Biot values, while the unsteadiness parameter shows a contrary response.
• The viscous-Ohmic dissipation tend to decrease the local Nusselt number.
• Enhancements in M_n_ and D_k_ lead to a reduction of the drag coefficient (skin friction coefficient).
• Streamlines are drawn to view the fluid flow over the surface in the presence of suction, injection, and absence of suction.
• The current model demonstrates remarkable effectiveness when using heat exchangers. Hybrid nanofluids (Ag-Gr/water) enhance the efficiency of heat transmission and optimize the thermal management of solar collectors.

**Table:** Nomenclature:

C_p_$\left(\frac{J}{kg.K}\right)$	Specific heat at constant pressure	M_n_	Magnetic Parameter
k $\left(\frac{W}{m.K}\right)$	Thermal conductivity	*Nu*	Nusselt number
$\mu \left(\frac{kg}{ms}\right)$	Viscosity coefficient	C_f_	Skin friction coefficient
B	Magnetic field strength	J_s_	Unsteadiness parameter
t (s)	time	Re	Reynolds number
$\delta \left(s^{-1}\right)$	Rate of stretching	E_k_	Eckert number
U_w_$\left(ms^{-1}\right)$	Velocity close to the surface	S_c_	Suction/injection parameter
$\sigma \left(\frac{S}{m}\right)$	Electrical conductivity	D_k_	Porosity parameter
$\rho \left(\frac{kg}{m^{3}}\right)$	Fluid density	Pr	Prandtl number
T_w_(K)	Temperature Near the Surface	B_t_	Biot number
h_0_$\left(\frac{W}{m^{2}}\right)$	Initial heat convection	$f^{\prime }\left(\eta \right)$	Dimensionless velocity
$T_{\infty }$ (K)	Ambient temperature	θ(η)	Dimensionless temperature
u, w $\left(\frac{m}{s}\right)$	Velocity components along the r and z direction	b	Positive constant

## CRediT authorship contribution statement

**M. Ragavi:** Writing – review & editing, Writing – original draft, Software, Methodology, Investigation. **P. Sreenivasulu:** Writing – review & editing, Validation, Supervision, Software, Methodology, Investigation. **T. Poornima:** Writing – review & editing, Writing – original draft, Visualization, Validation, Supervision, Methodology, Investigation, Conceptualization.

## Ethics approval and consent to participate

Not applicable.

## Availability of data and materials

On reasonable request, the corresponding author will provide access to the datasets used and analyzed during the present work.

## Funding

No funding

## Declaration of competing interest

We, the authors, hereby declare that the manuscript entitled “**Joule heating and dissipation couple effects on magneto silver-graphene hybrid nanofluids upon radial stretching surface** " is an original work and has not been submitted to any other journal for simultaneous consideration. The manuscript is not under review elsewhere and has not been previously published**.**

We confirm that all authors have read and approved the final version of the manuscript and agree with its submission to Materials Today Communications. The authors declare no conflict of interest.
